# Attention-deficit hyperactivity disorder drug search trends: a Scandinavian perspective

**DOI:** 10.1017/neu.2025.20

**Published:** 2025-07-11

**Authors:** Maximilian Zoltek, Richard Ågren

**Affiliations:** Department of Physiology and Pharmacology, Karolinska Institutet, Stockholm, Sweden

**Keywords:** Attention-deficit hyperactivity disorder, central stimulant, psychopharmacology, neuropsychiatry, Scandinavia, prescription patterns, Google Trends

## Abstract

Pharmacological treatment of attention-deficit hyperactivity disorder (ADHD) involves central stimulants and non-stimulant drugs. Because treatment preferences may vary geographically, we hypothesize that prescription data can be estimated from publicly available sources. First, we explore the relevance of internet search trends as proxies for real-life drug prescription patterns. Second, we identify geographical variations in ADHD drug trends over time. Publicly available Google Trends data for five ADHD drugs were analysed for the years 2010–2023. Temporal and spatial patterns were compared within Scandinavia, and the preference for central stimulants over non-stimulant drugs was compared across 17 countries. We find that internet search trends correlate with ADHD drug prescriptions. In the Scandinavian countries, a dominance of methylphenidate is observed, with rising internet search trends over time in Norway and Denmark. Furthermore, interest in lisdexamphetamine, relative to dextroamphetamine and atomoxetine, has increased sharply in recent years in the Scandinavian countries. The search proportion of central stimulants to non-stimulant drugs in Scandinavia ranges from 81% (Denmark) to 93% (Norway). Overall, internet search trends for ADHD drugs mirror reported prescription patterns and identify a dominance of methylphenidate, with an increasing interest in lisdexamphetamine. As such, search trends may serve as a feasible source for identifying geographical drug preferences.

## Graphical abstract

**Figure:**
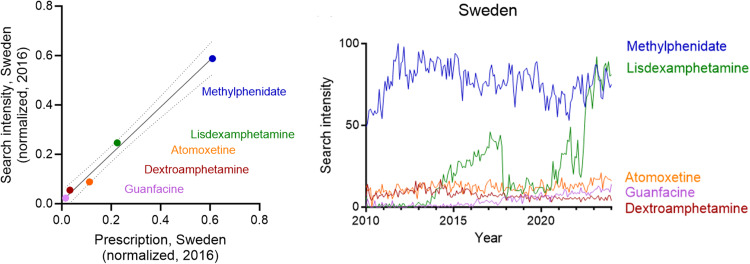

Significant outcomes

- Google search trends can be used as a proxy for ADHD drug prescriptions.
- Search trends indicate a recent increase in interest for lisdexamphetamine in Scandinavia.
- Globally, search trends suggest considerable variation in the prescription of central stimulants.

Limitations

- Internet drug search trends are non-specific and may be biased by non-medical attention.
- The used internet trend data are limited to relative comparisons.

## Introduction

Attention-deficit hyperactivity disorder (ADHD) is a neurodevelopmental condition that extends across the lifespan, imposing substantial impairment on affected individuals (Gallo & Posner, 2016). ADHD prevalence is homogeneous across continents, constituting approximately 5% of the population (Polanczyk *et al*., 2007; Polanczyk *et al*., 2015) and approximately 3.5% of the active labour force (de Graaf *et al*., 2008). Current scientific and medical consensus supports a neurodevelopmental origin of ADHD, which has shifted focus from behavioural interventions to pharmacotherapy (Posner *et al*., 2020; Kosheleff *et al*., 2023).

One of the first pharmacological interventions in treating ADHD was described in 1937, when academic performances of children with behavioural difficulties improved after daily amphetamine administration (Heal *et al*., 2013). Amphetamine is known to enhance noradrenergic and dopaminergic neurotransmission, elevating synaptic concentrations of monoamine neurotransmitters in the central nervous system (CNS; (Kuczenski *et al*., 1995; Heal *et al*., 1998; Thapar and Cooper, 2016). However, amphetamine suppresses appetite and fatigue and is associated with both psychiatric and somatic adverse side effects (e.g., anorexia nervosa, insomnia, hypertension, tachycardia, and motor symptoms) (James *et al*., 2001). Currently, amphetamine is approved by the Food and Drug Administration for ADHD. Methylphenidate, first synthesised in 1944, inhibits dopamine and norepinephrine transporters. However, unlike amphetamine, methylphenidate is reported to elicit its mechanism of action mainly via monoamine transporter inhibition rather than stimulating vesicular release (Shellenberg *et al*., 2020). To utilise the central stimulant effects of dextroamphetamine while managing the addictive risk, the prodrug lisdexamphetamine, with slower pharmacokinetics, was developed in the late 2000s (Heal *et al*., 2013; Swedish Medical Products Agency, 2022). Non-stimulant drugs include atomoxetine (a norepinephrine transporter inhibitor) and guanfacine (an adrenergic alpha 2A receptor agonist). In addition, off-label use of tricyclic antidepressants, clonidine, and bupropion constitute alternatives to central stimulants (Sharma and Couture, 2014).

In Sweden, methylphenidate constitutes the primary pharmacological treatment for ADHD among both children and adults. Second-line treatment includes amphetamine derivatives, with lisdexamphetamine preferred over dextroamphetamine due to a lesser risk of adverse effects (Swedish Medical Products Agency, 2022). Given the global prevalence of ADHD diagnoses and evolving treatment guidelines (de Graaf *et al*., 2008), we hypothesise that ADHD drug trends have shifted and may show noteworthy regional variation.

Multinational efforts have mapped differences in ADHD drug prescriptions in a few countries (Sørensen *et al*., 2023; Brikell *et al*., 2024). These investigations typically rely on retrospective registry-based data, which hinder geographical and temporal comparisons. Search intensity data from Google Trends have been shown to reflect drug prescription and epidemics (e.g., influenza and chicken pox) (Yang *et al*., 2015; Bakker *et al*., 2016; Lippi *et al*., 2017; Ågren, 2021). In this study, variations in search intensities are hypothesised to mirror geographical drug use patterns and policies. We leverage these trends to examine the ADHD drug landscape in Scandinavia and globally.

## Methods

### Google trends search data retrieval

**Figure 1.**
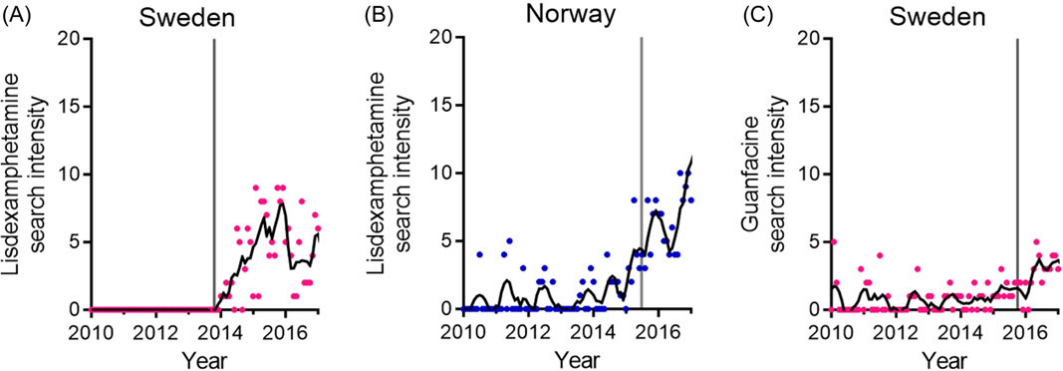
Temporal increases in search intensities coincide with drug approval. (A), (B) Lisdexamphetamine search intensities. Data from Sweden (A) and Norway (B). (C) Guanfacine search intensities in Sweden. Smooth polynomials of order 2 were adapted (black traces). The vertical lines denote drug approval time points for Sweden (A, C) and drug marketing time point for Norway (B).

**Figure 2.**
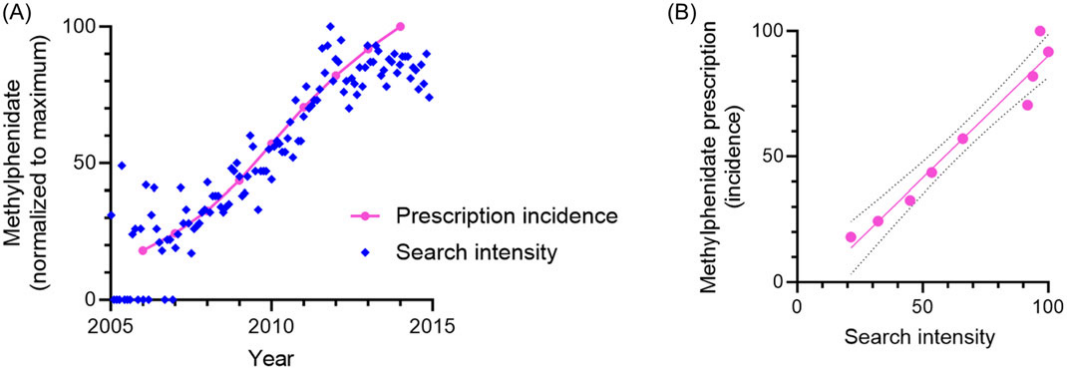
Methylphenidate prescription incidence is mirrored by internet searches. (A) Search intensity between the years 2005–2015 (blue) and prescription incidence (pink). (B) Correlation between prescription incidence and search trends, averaged per year. R^2^ = 0.95. Data from Sweden.

Publicly available internet search trend data were retrieved from Google Trends. Relative ADHD drug search intensities were compared between 17 countries (Japan, Germany, Mexico, Switzerland, Denmark, Finland, Sweden, Russia, Spain, Norway, Canada, United Kingdom, United States, Philippines, India, Netherlands, and Australia), with special emphasis on central stimulant drugs. This approach provides an observer-unbiased insight into ADHD drug trends (Fig. 1, fig. 2).

Google Trends provides relative temporal and spatial scores of up to five search indices (www.google.com/trends, accessed: 2024-02-01). The data sampling frequency depends on the time range (monthly data are available from January 2004). Relative search intensity measurements are presented as scores between 0 and 100. Five ADHD drugs – methylphenidate, lisdexamphetamine, dextroamphetamine, guanfacine, and atomoxetine – were included. All search terms were in the subcategory ‘drug’ or ‘substance’, referring to a specific substance in several languages. Temporal data for Sweden, Norway, Denmark, and Finland were retrieved for the period from January 2010 to December 2023. Methylphenidate search intensity data were retrieved starting from 2005 (see Fig. 2). Spatial data for all five drugs were retrieved for the full year 2023 for the 17 countries.

### Statistical analysis

Linear regression analysis was performed using GraphPad Prism 10. Correlations were assessed using Pearson’s correlation coefficient. For temporal data, second-order polynomial smoothing was applied to eight adjacent points along the *x*-axis.

## Results

### Internet ADHD drug search intensities correlate with prescriptions

First, we evaluate the search intensities of five ADHD medications over time. To assess whether these are temporally associated with prescription and availability, search intensities are associated with drug approval. For lisdexamphetamine, the search intensity rises after drug approvals in Sweden and Norway (Fig. 1(A) and (B)). Similarly, the guanfacine search intensity increases following drug approval in Sweden (Fig. 1(C)). Moreover, data on Swedish prescription incidence (Swedish National Board of Health and Welfare, 2016), here for methylphenidate between 2006–2014, correlate with search trend data for the corresponding period (Fig. 2 (A) and (B)).

**Figure 3.**
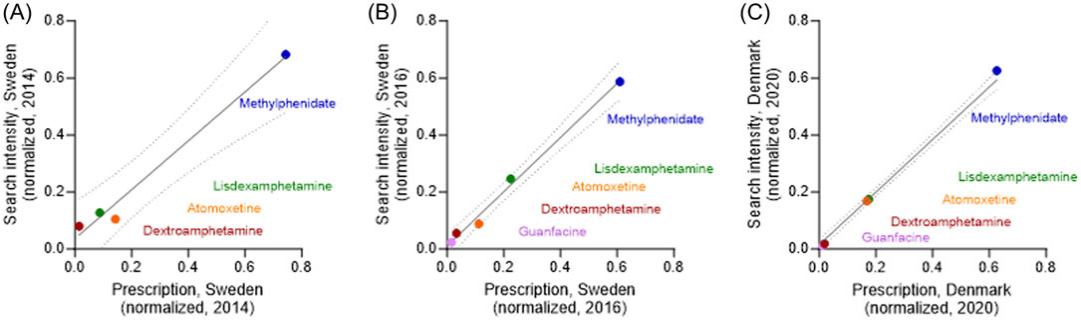
Correlation between search intensities and relative ADHD drug prescriptions. (A), (B) Data from Sweden from years 2014 (A) and until 2017 (‘2016’) (B). (C) Data from Denmark year 2020. Data are shown as fractions.

Next, we investigate how regional search intensities are associated with ADHD drug prescriptions. Normalised Swedish prescription data for years 2014 and 2016 (Swedish National Board of Health and Welfare, 2018) correlate with normalised search intensities (R^2^ = 0.98 and 0.99, respectively; Fig. 3 (A) and (B)). In similar, Danish prescription data from 2021 (The Danish Health Data Authority, 2021) associate with corresponding drug search trends (R^2^ = 0.99; Fig. 3 (C)).

### Dominance and increase of central stimulant search intensities in Scandinavia and Finland

**Figure 4.**
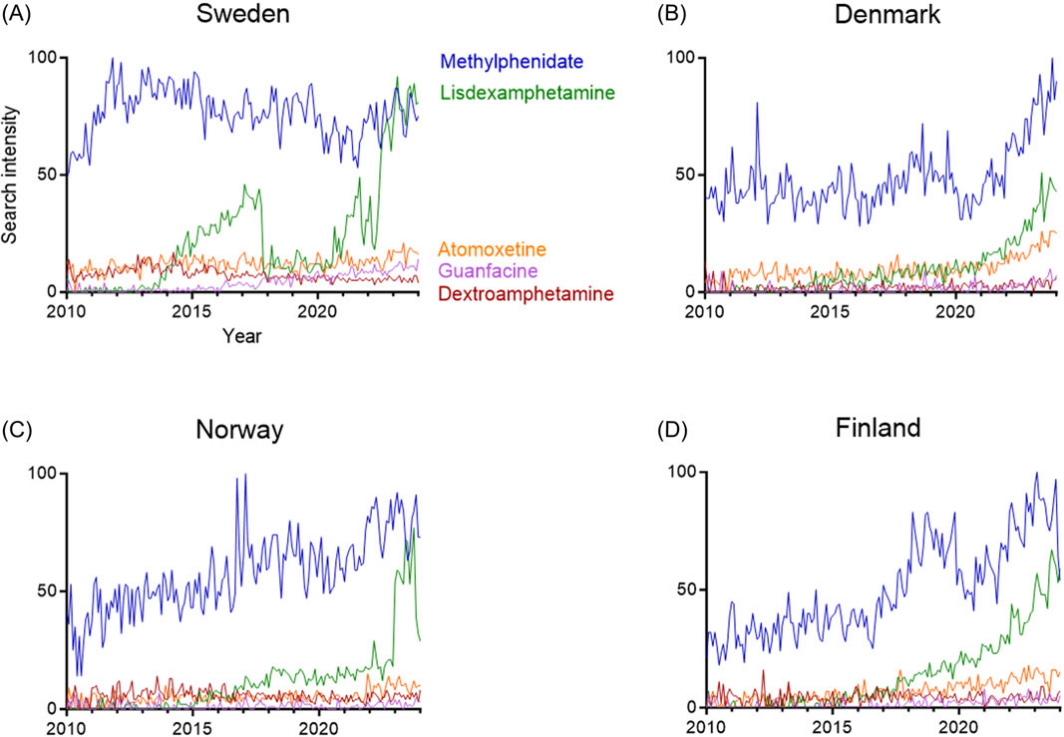
Country-based attention-deficit hyperactivity disorder drug search intensities in 2010–2023. Google Trends-based internet search activities for methylphenidate, lisdexamphetamine, atomoxetine, guanfacine, and dextroamphetamine. (A), (B), (C), (D) Temporal evolution in Sweden (A), Denmark (B), Norway (C), and Finland (D).

Given the temporospatial correlation between internet search trends and drug prescription over time and region, we next evaluate temporal trends of ADHD drug search intensities in Scandinavia and Finland. For these countries, methylphenidate is relatively dominant over the period 2010–2023 (Fig. 4 (A) and (D)).

Relative search interests for lisdexamphetamine increase from 2022 onwards (Fig. 4 (A-D)). Although guanfacine and dextroamphetamine search interests have remained stable and relatively low, the relative search intensities for atomoxetine have increased in Denmark during 2022–2024.

### Central stimulants are the most common ADHD drugs globally

**Figure 5.**
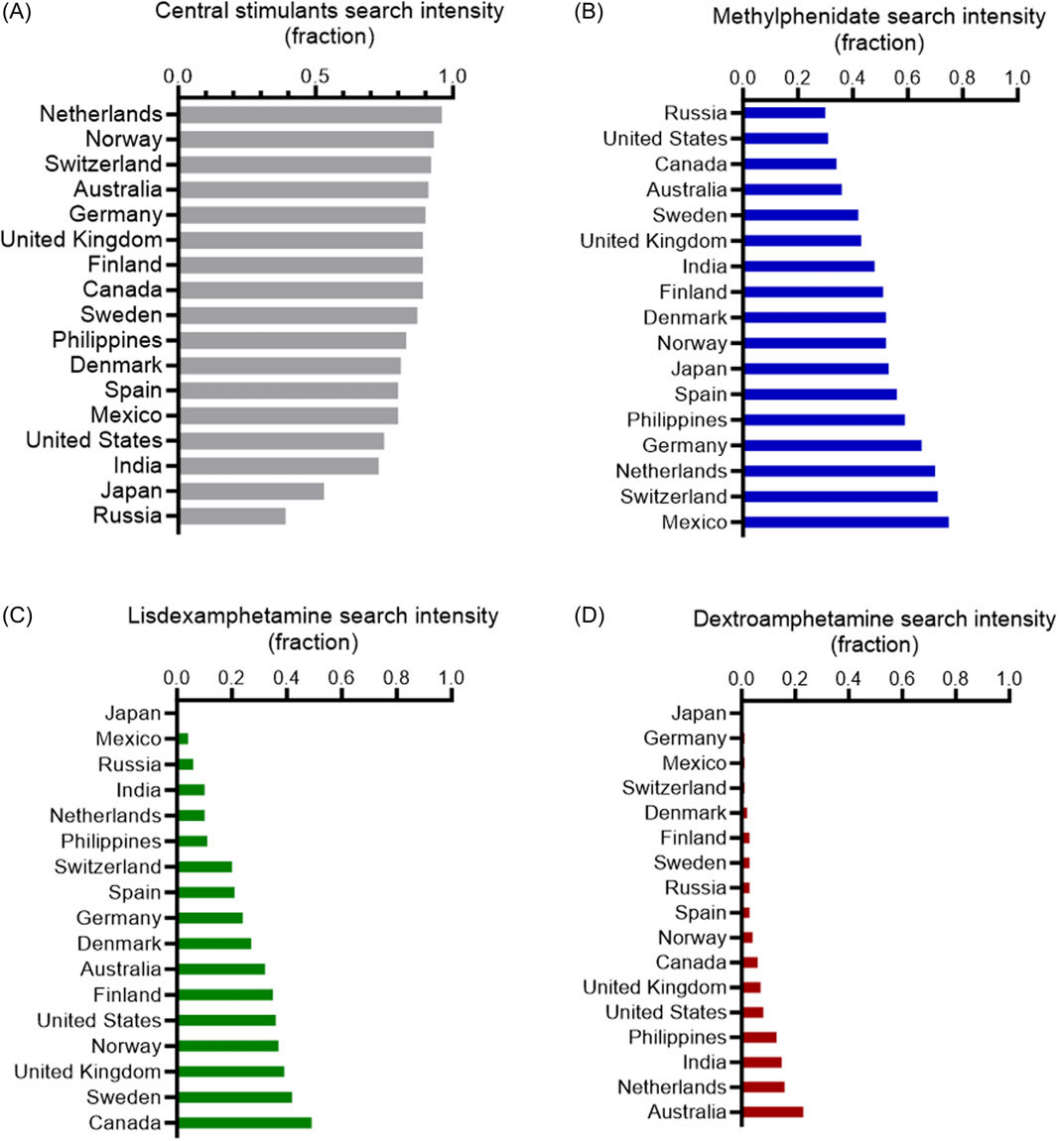
Relative central stimulant medication search intensities in 17 countries. (A) Pooled fractions of search intensities for methylphenidate, dextroamphetamine, and lisdexamphetamine relative to all five drugs (methylphenidate, dextroamphetamine, lisdexamphetamine, guanfacine, and atomoxetine). (B), (C), (D) Search intensities for methylphenidate (B), lisdexamphetamine (C), and dextroamphetamine (D) as a fraction of all five drugs. Data from 2023.

Next, we leverage the search trend algorithm to identify contributions of the three central stimulants to the ADHD drug search trends in 17 countries. We observe that central stimulants constitute 39–96% of the search intensities (Fig. 5 (A)). The Scandinavian countries demonstrate high fractions of central stimulant search trends; Denmark (81%), Sweden (87%), and Norway (93%) as a fraction of the five investigated ADHD drugs (methylphenidate, dextroamphetamine, lisdexamphetamine, guanfacine, and atomoxetine). Next, we investigate the relative contribution of the respective central stimulants. Methylphenidate search intensities of 30–75% are seen in all studied countries (Fig. 5 (B)), whereas lisdexamphetamine contributes to 0–49% of the central stimulant search intensities (Fig. 5 (C)). For dextroamphetamine, the variation in search intensities is 0–23%, with the highest values observed in Australia (Fig. 5 (D)).

## Discussion

In recent years, there has been a noticeable increase in the incidence and prevalence of ADHD, both in Scandinavia and globally (Posner *et al*., 2020; Sørensen *et al*., 2023). Using a digital epidemiology approach that leverages publicly available temporal and spatial internet search query data, we investigated public interest in ADHD medications. First, we validated this method by demonstrating that internet search intensities correlate with corresponding drug prescriptions over time. Subsequently, utilising this open-source approach, we observed relative increases in methylphenidate prescriptions in Norway, Denmark, and Finland during the study period. In recent years, we also observed a surge in interest in lisdexamphetamine. These trends in Scandinavia align with findings from contemporary population-based studies in nine countries (Brikell *et al*., 2024). Similarly, the relatively high search interest in dextroamphetamine in Australia corresponds to reported increases in prescriptions (Hollingworth *et al*., 2011; Brikell *et al*., 2024).

The relatively low search intensities for guanfacine may reflect factors such as clinical guidelines, availability, or the drug’s effects. Although guanfacine has been shown to be effective in treating ADHD symptoms compared to placebo (Yu *et al*., 2023), its efficacy in children and adolescents appears to be on par with methylphenidate but less effective than amphetamines. However, there is insufficient data to draw definitive conclusions about its efficacy in adults. In terms of tolerability, amphetamines and guanfacine were found to be less well tolerated than placebo in children. Similar patterns of tolerability have been observed in adults, particularly for methylphenidate, amphetamines, and atomoxetine (Cortese *et al*., 2018).

Selection of ADHD medication is likely influenced by a variety of patient-specific and general factors, of both medical and non-medical character. In Scandinavia, the structure of public healthcare systems, combined with national treatment guidelines and broad availability of medications, may impact the choice of first-line treatment (Ludvigsson *et al*., 2009).

The correlation between search intensities and ADHD drug prescription, as demonstrated by a) trend increases after approval, b) temporal associations, and c) relative drug preferences, supports the methodology used in this study. However, it is important to note that the analysed search intensities do not fully reflect true prescription rates or usage. Instead, they capture a combination of interest from patients, healthcare professionals, researchers, and the public. Factors such as media bias and bursts of public interest unrelated to medical prescriptions can influence these findings. Moreover, discrepancies between search intensities and prescription data may arise from factors such as restricted access to or awareness of Google resources, or differences in prescription data between public and private healthcare systems. Additionally, Google Trends does not disclose specific details about the absolute search intensities, underlying calculations, or inclusion thresholds, so the data presented here are reported in relative terms. A similar methodology has been evaluated for a wider range of drugs, regions, and longer time periods (Ågren, 2021).

Conclusively, internet search trends for ADHD drugs closely mirror reported prescription patterns. Temporal and spatial assessments of ADHD drug preferences in Scandinavia highlight a dominance of methylphenidate and show an increasing interest in lisdexamphetamine. Internet search trends may provide a current view of ADHD drug preferences and use.

## Data Availability

All data generated and analysed during this study are included in this published article (and its Supplementary Information files).
